# Engaging primary care professionals in collaborative processes for optimising type 2 diabetes prevention practice: the PREDIAPS cluster randomised type II hybrid implementation trial

**DOI:** 10.1186/s13012-018-0783-0

**Published:** 2018-07-11

**Authors:** Alvaro Sanchez, Gonzalo Grandes, Susana Pablo, Maite Espinosa, Artemis Torres, Arturo García-Alvarez, on behalf of the PREDIAPS Group, Amaia Bengoetxea, Olga Galarza, Elsa Martínez, Itziar Zalduegi, Dorothea Chausson, Agurtzane Gorroño, Alicia Pollán, Marisol Bernabéu, Ma Yolanda Calvo, Ander Artiagoitia, Nerea Zaramillo, Lidia Gonzalez, Asier Aurrekoetexea, Jon Azkarate, María Muñoa, Mar Bilbao, Vicki Camineiro, Gonzalo Gómez de Iturriaga, Fernando Gago, Iciar Ochoa de Retana, Ana Zorrilla, Ma Luisa Gutiérrez, Jone Capetillo Serra, Ma Nieves Lopez, Nekane Iguerregui, Dolores López, Maite Gastañaga, Antonia Flores, Marcos Pereda, Amaya González, Ana Castresana, Laura Saiz, Nerea Regulez, Estibaliz Peciña, Jasone De la Plaza, Lucía Irastoza, Jose Contreras, Idoia Etxebarria, Begoña Oleaga, Cristina Herrero, Nora Cabezón, Fátima Calvo, J. Manuel Llamazares, Ma Ángeles Gutierrez, Monica Prieto, Concepción Estébanez, María José Cordovilla, Alicia Domínguez, Isabel Lázaro, Elena Resines, Yolanda Villalba, Begoña Ayerdi, Florencia Martín, Magdalena Presmanes, Floreal Crespo, Araceli Benito, Ma Belén Molina, Ma Mar García, Ma Gracia Díaz, Ma Luisa Rodriguez Ortiz de Zarate, Rebeca San Cristóbal, M. Zugazaga Prieto, Pedro Martínez, Mercedes Crespo, Estíbaliz Albitre, Adelina García-Roldán, Amaia García, Maite Castro, Iñaki Gorospe, Amelia V. Hernández, Maite López, Mirian Sainz, Irene Marín, María Jesús Aragón, Leire Ulayar, Encarnación Santamaría, Carmen Sánchez, Javier Bayo, Begoña Urkullu, Ana Inés Pereda, Mercedes Garcia, Pilar Blanco, Silvia Soler Valverde, Jose I. Atela, Hiart Trespalacios, Anabel Llarena, Verónica Ruiz, Begoña Cabieces, Concepción Ugarte, Guadalupe Icaza, Edurne Zubeldia, Idoia González, Ángeles Gayo, Itxaso Arévalo Martín, Gloria Intxausti, Esther García, Teresa Sánchez, Igone Lobato, Noelia Fuente, Naiara Ortolachipi, Edelweiss Sánchez, Victoria Cosgaya, Ángeles Gayo, Arantza Azazeta, Patricia Zaballa, Ana Isabel Ramila, Teresa Rodeño, Inmaculada Rodríguez, Teresa Vázquez, Raquel Ruíz, Rosa Herrero, María Valvanera Aduna, Saioa Setién García, Begoña Ruíz, Juan José Casas, Joana Clemente, Javier Amiama, Javier Angulo, Belén Aramendia, Soledad Asenjo, Ma Sonia Mayoral, Remedios Oyarzun, Bergoi Calvar, Belinda Zulueta, Edurne Elola, Josu Egaña, Gemma Díaz, Ma Ángeles Sola, Laura Balague, Ma Ángeles Ganzarain, Arantxa Aramburu, Ana Ma Guine, Edurne Lizarazu, Inma Valverde, Nekane Arenas, Susana Alonso, Rosa Salaberria, Javier Merino, Mercedes Álvarez, Ester Lázaro, Juncal Izcara, Leire González, Ainhoa García Leunda, Idoia Sánchez, Esther Usandiaga, Eluska Yetano, Jaione Larrea, Inés Mendinueta, Asún Uria, Ana Belén Gaztañaga, Eva Mayo, Onintza Aranzadi, Eulalia Medina, Rosa González, Ione Gutiérrez, Arantxa Perez, Ma Ángeles Izquierdo, Alejandro García, Ainhoa Ugarte, Ma Teresa Zubeldia, Bingen Uriondo, Ma Carmen Aranegui, Arantxa Mendiguren, Yolanda Fernández, Maite Zapirain, Ma Jose Garín, Aitziber Ayerbe, Jon Urkia, Alvaro Sánchez, Josep Cortada, Esther Gorostiza, Maite Espinosa, Susana Pablo, Artemiss Torres, Gonzalo Grandes, Alicia Cortazar, Virginia Bellido, Patxi Ezkurra, Rafa Rotaetxe

**Affiliations:** 1 Primary Care Research Unit of Bizkaia, Basque Healthcare Service, Osakidetza, BioCruces Health Research Institute, Luis Power 18, E48014 Bilbao, Spain; 2 Osakidetza, Unidad de Investigación de Atención Primaria, Luis Power 18, 4ª planta, E-48014 Bilbao, Spain

**Keywords:** Pre-diabetes, Primary health care, Prevention, Cluster clinical trial, Implementation strategy, Interprofessional collaborative practice

## Abstract

**Background:**

There is a lack of evidence concerning the effectiveness of different strategies to engage healthcare professionals in collaborative processes that seek to optimise clinical practice. The PREDIAPS project aims to assess the effect of different primary health care (PHC) providers’ engagement procedures in the creation and execution of a facilitated interprofessional collaborative process to optimise the integration of the recommended clinical practice for the prevention of type-2 diabetes (T2D) in routine PHC.

**Methods:**

This will be a randomised cluster type II hybrid implementation trial. Nine PHC centres from the Basque Health Service (Osakidetza) will be allocated to two different procedures to engage family doctors and nurses and create an interprofessional collaborative practice to optimise the integration of a T2D primary prevention programme. All centres and PHC professionals will receive training on current guidelines in primary prevention of T2D and effective interventions to promote healthy lifestyles. Headed by a local leader and an external facilitator, centres will conduct a collaborative structured process to model and adapt the intervention and its implementation to the specific context of professionals and centres. One of the groups will apply this strategy globally, promoting the cooperation of all health professionals from the beginning. The other will perform it sequentially, centred first on nurses, who will then seek the pragmatic cooperation of doctors. All patients without diabetes aged ≥ 30 years old who attend collaborating centres at least once during the study period and found to be at high risk of developing T2D will be eligible for programme inclusion. The main outcome measures focus on changes observed in indicators of T2D prevention clinical practice at centre level after 12 and 24 months, associated with the application of one or other engagement procedure. Secondary outcomes will compare their clinical effectiveness in changing eligible exposed patients’ main lifestyle behaviours and risk factors (physical activity and diet, weight, etc.) after 12 months.

**Discussion:**

The PREDIAPS project will generate scientific knowledge on procedures for engaging PHC professional to facilitate feasible and effective adoption of proven interventions for the prevention of T2D in routine clinical practice through the application of implementation strategies.

**Trial registration:**

Clinicaltrials.gov identifier: NCT03254979. Registered 16 August 2017.

## Background

The dramatic increase in the rates of type 2 diabetes mellitus (T2D) and associated complications has made it a major health problem worldwide [1]. Despite the growing evidence provided by landmark T2D prevention trials and several translational studies assessing both the efficacy and effectiveness of healthy lifestyle prevention interventions in clinical and community settings [2–12], the actual translation and integration of primary prevention programmes in routine clinical practice remains an unsolved challenge. Indeed, currently, in the real context of the provision of services seeking to prevent T2D in the Basque Health Service (Osakidetza), only 30% of patients at high risk of developing this disease receive some type of intervention focused on preventive advice.

In the PreDe multicentre trial conducted in 14 Osakidetza PHC centres, it was shown that an intervention promoting healthy lifestyles among patients at high risk of developing the disease succeeded in reducing the incidence of T2D by almost a third (32%) [12]. Nevertheless, despite having identified an intervention that is potentially effective for preventing T2D in PHC, the passive implementation strategy used for guiding the translation and adoption of the intervention in a routine clinical context by our health professionals has not achieved feasible and sustainable deployment, with low levels of adoption by clinicians, limited population coverage, failure to integrate the intervention into practice, and lack of continuity at the end of the project, among other problems. The factors identified by the professionals as the main barriers that constrained the feasibility of integration of the programme under routine conditions in the centres and implied that it would not be sustainable were the lack of participation and commitment from the staff of the centres in the context of time pressures and heavy workloads. These two factors, in turn, limited the organisation of operational elements required for putting the programme and its components into practice, in an environment with few incentives and an overload of corporate initiatives [13].

Numerous strategies for changing clinical practice have been tested with variable degrees of success. There is a consensus in the scientific literature that passive learning or dissemination strategies are ineffective [14]. In contrast, key elements in the success of implementation strategies seeking to optimise clinical practice include internal leadership to drive change and implementation [15, 16], active and participatory engagement of professionals in collaborative processes [17–19], external facilitation [20, 21], adaptation of interventions to the local context and its determinants [22, 23], and the combination of multiple components [14]. Among these effective elements, one of the areas for which there is still a lack of clear evidence concerns the effectiveness of strategies for engaging professionals in the aforementioned collaborative innovation processes [19, 24–26]. It is not known what factors are essential for effective interprofessional collaboration, especially in the real-world of PHC, with the overall context of overcrowding and work overload, and marked differences by professional category, both in terms of identity and competencies [19].

Interprofessional collaboration (communities of practice, work teams or collaborative interprofessional processes) has long been considered an essential factor underpinning effective PHC [27]. In general, the practice of interprofessional collaboration characterised by mutual respect and trust, open communication between individuals, holding a shared vision of the goals and potential benefits of the intervention, as well as joint decision-making by consensus on how the intervention should be implemented, has been shown to improve clinical practice and health outcomes [15, 19, 20, 28–30]. Yet, the mere placing of healthcare professionals with different backgrounds in a team does not mean that they will have the knowledge or skills necessary to work together and collaborate. Further, there is evidence that the effective implementation of change in organisations is often obstructed by organisational and professional demarcations that hinder the sharing of knowledge and dissemination of working practices [25, 31-33]: professional differences may have a dual effect leading, on the one hand, to innovation, learning and cross-fertilisation between practices, but on the other, to division, fragmentation and disconnection. Great efforts are needed to overcome such professional boundaries and create functional interprofessional collaboration, given its complexity [19].

Research on interprofessional work teams and collaborative communities of practice establishes that collaboration is an interpersonal process that requires joint engagement in intellectual activities and that interaction between members of the team, concerning the assignment of functions and limits between professionals, hinge on two principal concepts: independence and collaboration [34–36]. Collaboration is an interpersonal process implying joint participation in intellectual activities, while independence is associated with independent self-determined practice [36]. These concepts of collaboration and independence have emerged as complementary factors that may improve the provision of health services, though in extreme cases, they may disrupt the work of a team. Based on theoretical models of the engagement of health professionals and research in the field, a spectrum of strategies for engagement in cooperative practices that favour interaction between different types of professionals have been identified, ranging from not promoting any type of relationship or engagement—“Individualism”, through encouraging some degree of closeness between groups—“Creation of relations”, to affirming a common identity that includes all the groups—“Promotion of a common identity” [26]. Hence, on the basis of the few studies conducted in this field, it seems that independence may be an important element in the functioning of an interprofessional team. The empowerment of members of the team to develop their independence may improve collaborative interactions. While an ability to swap roles and functions may help to reduce the work load of group members, it may also increase the level of conflict related to fighting for power as the roles of the different professionals are less well defined [35].

In short, the engagement of health professionals is a crucial factor in the processes of change and optimisation of clinical practice. Nevertheless, we do not know what is the best procedure for creating the aforementioned interprofessional teams or communities of practice in PHC and ensure that they produce effective and functional collaborative interaction, in a shared framework for learning and practice based on differentiation rather than fragmentation by professional group [19, 24, 25, 35]. We need a better understanding of how to establish and manage teams or collaborative communities of practice with a view to maximising their potential to improve professional development and healthcare, change practice or implement evidence-based practice.

The objective of the present study will be to assess the effect of different strategies for generating engagement of PHC professionals in the creation of an interprofessional community of practice and setting up of a structured collaborative modelling process headed by an external facilitator and a local leader, in order to optimise clinical practice concerning the primary prevention of T2D in the routine clinical context of PHC services in Osakidetza. The implementation strategy will be the same in both groups and will contain elements shown to be key factors in successful optimization of clinical practice: leadership, facilitation, engagement of professionals, and adaption to the local context, among others. The comparison groups will differ, however, in the order of activation of professional categories: a sequential strategy of starting with nursing staff and eventually involving family doctors, versus a global approach seeking the participation of all the professionals from the outset. It is hypothesised that the sequential strategy will be more effective for accelerating the adoption of recommended clinical interventions and ensure that they are maintained, as initially it will be less complex, and more flexible and adaptable to the local context, and hence can be expected to generate less conflict between professionals. Further, from the outset, there will be a focus on a clinically effective intervention (intensive intervention in the promotion of healthy lifestyles), carried out by nurses and later seeking the cooperation of other professionals, once it has been shown that the nursing staff are actively committed to change in health promotion practice.

Specific objectives are the following:

1. To estimate the effectiveness attributable to the strategy for engaging health professionals in the improvement of the clinical practice for preventing T2D in high risk individuals, comparing the following implementation indicators between the centres assigned to the sequentially facilitated collaborative model strategy and those assigned to the globally facilitated collaborative model strategy.
2. To estimate the potential clinical effectiveness of the intervention programmes comparing the proportion of users who change their lifestyle habits (physical activity and diet) and lose 5% of their body weight (a) observed in the population exposed (pre-post comparison) in both groups and (b) comparing the figures obtained in the programmes following the sequential vs the global strategy.
3. To analyse the factors associated with successful implementation results in the implementation strategy in general and in the engagement procedures assessed in particular, at the organisational collaborating health professional and exposed patients levels.
4. To explore the perceived feasibility and satisfaction of health professionals and users exposed with the implementation processes and the intervention programmes.

## Methods

### Design

This is to be a multicentre randomised type II hybrid implementation trial of feasibility and potential effectiveness in nine primary care centres that will be randomly allocated to one of the two strategies for engaging healthcare professionals in the development and execution of a process for adopting the recommended clinical practice for T2D prevention: a global strategy, seeking cooperation between professionals at all levels from the outset; or a sequential strategy, first led by nurses, and then seeking the pragmatic cooperation of the rest of the professionals in the centres. The research protocol has been approved by the Basque Country Clinical Research Ethics Committee (Ref. no.: 08/2015).

### Participants

#### Primary care centres

Nine PHC centres of Osakidetza will be included in the study based on their organisational willingness to optimise the primary prevention of T2D, assessed through the obtaining of written commitment and consent to collaborate from a majority (> 51%) of the centre’s nurses and a substantial proportion of the doctors whose patients’ lists are involved through the nursing staff, after a session presenting the project.

#### Users of primary care services

All the patients aged between 45 and 70 years old who seek medical attention in the participating centres at least once with a high risk of developing T2D and/or with moderate hyperglycaemia (high baseline glucose levels or glucose intolerance), but no diagnosis of T2D documented the medical record, will be candidates to participate in the T2D prevention programme.

### Recommended clinical intervention based on evidence and clinical practice guidelines

Based on the available scientific evidence and clinical practice guidelines [2, 4, 13], three measures are recommended for the prevention of T2D in clinical settings:

(1) Screen for individuals with abnormal regulation of glucose or a high risk of developing T2D (fasting glucose ≥ 110–125 mg/dl, HbA1c ≥ 6%), with the following approaches being recommended: T2D screening as part of opportunistic screening for cardiovascular risk in individuals ≥ 45 years old or ≥ 30 years old with at least one known risk factor (e.g. hypertension); T2D screening based on a body mass index (BMI) ≥ 25 kg/m^2^ in individuals between 40 and 70 years old; or screening for T2D risk using the FINDRISC [13] scale in individuals between 45 and 70 years old. These last two procedures should be completed with a fasting glucose to discard T2D diagnosis.

(2) In patients identified as high risk, carry out a structured intensive programme focused on the prescription of personalised plans for lifestyle change (low-calorie and low-fat diets, and at least 150 min of exercise per week). This intensive intervention requires the assessment of lifestyle habits, personalised advice (tailored to the individual’s risk) and the setting of objectives and plans or prescription of a personalised intervention for lifestyle change. It should combine education with the use of theoretically-grounded evidence-based strategies for behavioural change and the introduction of changes in daily life.

(3) Ensure that the intervention is followed up to keep participants motivated, with frequent contact at the beginning and subsequently annual follow-up.

### Random allocation

The implementation strategy to facilitate the adoption of the recommended clinical intervention for T2D prevention will be common to the two comparison groups (see the following section). In order to distinguish and isolate any effects of the strategy used for the creation of the multidisciplinary community of practice, the centres will be randomly allocated to one of the comparison groups: five centres to the sequential strategy and the other four to the global strategy. This random allocation will be carried out by the members of our research unit not involved in this project, using a computer-generated random number sequence obtained before starting the trial. Given that the new programme is an optimisation of T2D prevention activities already offered by the Basque Health Service, it is expected that participating patients will be blinded to group allocation.

### Implementation strategy

The implementation strategy to facilitate the adoption of the recommended intervention in clinical practice was developed in three main steps:

1. First, we analysed the factors or determinants that have played a key role in the feasibility of setting up a primary prevention programme for T2D in the context of a previous clinical trial, the PreDe trial [13]. For this purpose, the health professionals of the participating centres carried out a three-part structured process: (a) individual identification of the main barriers and facilitators in putting the intervention programmes into practice; (b) individual rating of the importance or impact of each factor identified; and (c) pooling and agreed prioritisation of the factors using a nominal group technique, that resulted in a priority list of determinants/targets for improvement of T2D prevention in PHC.
2. In a second step, we designed a tailor-made strategy to address the aforementioned determinants related to clinical practice for T2D prevention, on the basis of theoretical implementation frameworks. For this, each of the determinants identified was framed in one of the possible implementation constructs/determinants and subsequently one or several specific techniques or strategies for overcoming/strengthening the barriers/facilitators in accordance with theoretical frameworks for implementation, the Consolidated Framework for Implementation Research (CFIR), and Theoretical Domains Framework (TDF), and a compendium of implementation strategies [23, 37, 38].
3. Based on previous experience [39–42], the research team involved in the implementation organised and operationalised the specific strategies into actions to be put into practice in the health centres in a framework of a dynamic implementation process.

The same multifaceted implementation strategy will be applied in all the centres, yet the process for establishing the community of practice will differ (Table 1). The first step of the process is the consensus-based selection in each centre of the local leader and his/her subsequent training (15 h over three sessions), organised by comparison group (global or sequential). The first session will have the same content for both groups and will seek to enable the local leaders to acquire the necessary knowledge on primary prevention of T2D and the recommended clinical intervention. The second session will address aspects of the assessment of the organisational and inter-personal structure of the corresponding centre as well as communication and leadership techniques and skills. The third session will be focused on drafting the implementation plan and will address the strategies and procedures to be used in each group. Subsequently, we will provide theoretical and practical training related to the evidence-based clinical intervention for the primary prevention of T2D (90 min) and the computer support tool (6 h). The training will consolidate the differences between the strategies, in that in the sequential group only the nurses will initially receive this training, training for doctors being postponed until after the planning and the carrying out of the cycles of piloting.

**Table 1:** Outline of implementation actions by comparison group

**Implementation actions: goals and concrete strategies**	**Comparison Groups (Target agents)**	
**Facilitated community of practice**		
*Goals* *: to facilitate a collaborative learning environment to improve the implementation of clinical innovation.* *Concrete implementation strategies:* *Create a learning collaborative, Facilitation in the form of outreach visits*	**Global** *Intergroup partnership* and *Relationship building*	**Sequential** *Pragmatic cooperation*
**Strengthening of Local Leadership** *Goals* ***:*** *to provide the local coordinator with skills in primary prevention of T2D and interpersonal and organisational strategies to support the implementation.* *Concrete implementation strategies* *: Recruit, designate and train for leadership; Educational meetings.*		
**3 training sessions** (5 hours/session)	Local leaders for global strategy	Local leaders for sequential strategy
**Training in clinical intervention** *Goals* *: to provide initial training in recommended effective clinical intervention for the prevention of T2D and in how to use the information technology support tool in the electronic health record.* *Concrete implementation strategies* *: Educational and capacitation meetings; Changes in record systems*		
**Session 1**: Primary prevention of T2D in PHC: evidence and recommended practice (90 min) **Session 2**: Computer application for the promotion of healthy habits in the Electronic Health Record (6 hours)	Doctors and Nurses	Nurses
**Collaborative structuring of the programme** Goals: to plan the local programme based on shared decision-making concerning: objectives, actions, agents, work flow, organisation and sharing out of tasks. *Concrete implementation strategies: Local needs assessment; Educational and outreach meetings eliciting local consensus discussion; Ongoing training; Cyclical small tests of change; Develop a formal implementation blueprint*		
**Session 3** – Needs assessment and prioritisation of areas for improvement in T2D prevention (90 min)		
**Session 4/5** – Planning T2D prevention programme (180 min) **Session 6**: Plan-Do-Study-Act cycle 1 (90 min)		Nurses (*Prescription→Screening*)
**Session 7**: Plan-Do-Study-Act cycle 2 (90 min)	Doctors and Nurses (*Screening→Prescription*)	
**Session 8**: Refresher training (180 min)		Doctors and Nurses (*from the 2*^*nd*^ pilot*)
**Session 9:** Plan-Do-Study-Act cycle 3 (90 min)		
**Session 10**: Final standardisation of the local T2D prevention programme (90 min)		
**Ongoing sustainability** *Goals* *: to continually support and assess innovation being put into practice.* *Concrete implementation strategies* *: Develop quality monitoring systems; Audit and provide Feedback; Ongoing training*		
Regular audits and ongoing facilitation: 6 follow-up sessions over the course of 12 months (90 min X 6 sessions) Continuing education in clinical intervention and information technology tools	All participating professionals	

*A more extensive session lasting 120 min is required to share information about the preliminary programme, suggest pragmatic cooperation and organise the second pilot (2^nd^ PDSA)

Bolded text refers to an implementation action (composed of multiple concrete implementation strategies responding to a implementation goal) or to the work sessions to be held within an implementation action

Underlined text refers to implementation goals or concrete implementation strategies pertaining to an implementation action

Once the training and skill acquisition phase is over, we will start the collaborative modelling process, based on the establishment of a community of practice headed by a local leader and an external facilitator. In the global strategy group, this will be undertaken jointly by participating doctors and nurses from the outset, seeking to adapt the set of clinical activities in a logical way: screening for risk (selecting from among the recommended approaches), gathering of information on individuals’ habits, provision of advice, and intensive intervention with prescription of personalised plans for lifestyle change. In this way, it is hoped that a community of practice will be fostered based on an approach that lies between “Intergroup partnership” and “Relationship building” [26], underlining collaboration between professional categories from the beginning [36]. In the sequential group, first the nurses will consolidate the execution of the clinical intervention at their level, working through from the last “active ingredient” to the first, that is, from the prescription of personal plans to screening. Subsequently, they will involve doctors in the framework of a community of practice underpinned by “Pragmatic cooperation” [26], underlining the independence of each professional category [36].

Once a preliminary programme for primary prevention of T2D has been planned, we will move onto a process of brief pilots of specific actions to assess their efficiency and, thereby, optimise the set of actions of the programme. In the global group, doctors and nurses will collaborate in the piloting process (with joint participation across the professional categories). On the other hand, in the sequential group, once the clinical intervention has been started by nurses, the first cycle of piloting will be undertaken. After this, pragmatic cooperation of doctors will be sought to maximise the efficiency of the actions, flows and procedures of the intervention programme, doctors being involved in the second cycle of piloting. The collaborative process is completed with a further training on the clinical intervention and the IT support tool (this being the initial training of doctors in the sequential group), a third piloting cycle to finalise the details and final standardisation of the programme. The aim of this standardisation is to establish and set the standards for practice, the target population for the intervention and screening strategy, and key components of the intervention and follow-up: broken down by action and process, staff involved, material and organisational components, and factors specific to the context of the centre, among others. The programmes will then be incorporated to the portfolio of services of the centres.

The last element of the strategy is follow-up or sustained support, which will involve six follow-up sessions over 12 months (every month for the first 3 months, then at 6, 9 and 12 months) and the use of auditing reports, presented jointly by the local leader and the external facilitator.

### Assessment of results

1. Main outcome measures: effectiveness in the optimisation of clinical practice for the primary prevention of T2D (experimental comparison) (see Table 2 for a detailed description):
  The proportion of professionals who actively adopt the intervention programme, out of the total in each centre (adoption)
  The proportion of individuals in whom clinical practice recommendations on the screening for T2D risk are met out of the total number of potentially eligible individuals seeking medical care (reach and coverage)
  The proportion of non-diabetic patients at high risk of developing T2D exposed to interventions related to lifestyle habits 12 months after the introduction of the intervention programmes: assessment of lifestyle habits, provision of personalised advice, prescription of lifestyle changes and follow-up (execution of the recommended clinical intervention).
2. Secondary outcome measures: clinical effectiveness of the intervention (observational comparison)

**Table 2:** Main outcome measures

**Effectiveness in the optimisation of clinical practice for the primary prevention of T2D (experimental comparison)**	
**Measures**	**Timing and Source**
**Coverage**: Screening indicators (depending on the screening strategy selected by the centre)	
*Case 1* *: T2D screening as part of opportunistic screening for cardiovascular risk in individuals aged ≥45 years or ≥30 years with at least one known risk factor:* • % of non-diabetic patients aged ≥30 years with a cardiovascular risk factor (e.g., hypertension or BMI ≥30 kg/m2 or hyperglycaemia) attending to family doctor office in whom clinical practice guidelines are followed in terms of T2D screening using fasting glucose levels in the previous year; • % of non-diabetic patients aged ≥45 years with no cardiovascular risk factor attending to family doctor office in whom clinical practice guidelines are followed in terms of T2D screening using fasting glucose levels in the previous 4 years.	
*Case 2* *: T2D screening based on a BMI≥25 kg/m2 in individuals aged 40 to 70 years:* • % of non-diabetic patients aged between 40 and 70 years attending to family doctor’s office whose BMI has been measured in the previous year, 12 months after the introduction of the T2D prevention programme in the centres;	Baseline and 12 months after the setting up of the programme
• % of non-diabetic patients aged between 40 and 70 years attending to family doctor’s office with a BMI≥25 kg/m2 who have been screened for T2D using fasting glucose in the previous year, 12 months after the introduction of the T2D prevention programme in the centres.	Electronical Health Record
**Implementation**: Execution of the elements of the intervention programme in high risk patients defined by the presence of prediabetes (fasting glucose 110-125 mg/dl)	
•% of patients whose physical activity levels and diet have been assessed, after the identification of T2D risk; •% of patients who have been given preventative advice concerning the need to increase physical activity and eat a healthy diet, after the identification of T2D risk;	
•% of patients who have been prescribed a plan for increasing physical activity and eating a healthy diet, after the identification of T2D risk;	Baseline and 12 months after the setting up of the programme
•% of high risk patients (fasting glucose 110-125 mg/dl) who have undergone annual testing of fasting glucose and HbA1c.	Electronical Health Record
**Maintenance:** Long-term execution of the healthy lifestyles promotion programme in T2D high risk patients	
•Level of screening coverage among candidate patients (e.g., % of patients with screening for T2D) •Level of execution of the clinical intervention elements (e.g., % of pre-diabetic patients who have received a prescription for lifestyle change) 24 months the introduction of the programme	0 to 24 months after the setting up of the programme (monthly rate)
•Monthly rate of the change in the coverage and execution of intervention elements for the promotion of healthy lifestyles over a 24-month period.	Electronical Health Record
**Spreading:** healthy lifestyles promotion actions in attending patients who do not meet the criteria of high risk of T2D (e.g., overweight or obese patients with normal glucose levels).	
•% of patients whose physical activity levels and diet have been assessed, from those attending aged 10 to 80 years;	
•% of patients who have been given preventative advice concerning the need to increase physical activity and eat a healthy diet, from those attending aged 10 to 80 years;	Baseline, 12 and 24 months after the setting up of the programme
•% of patients who have been prescribed a plan for increasing physical activity and eating a healthy diet, from those attending aged 10 to 80 years;	Electronical Health Record
**Secondary outcome measures: clinical effectiveness of the intervention (observational comparison)**	
**Change in healthy lifestyles and cardiovascular risk factors** of high risk patients exposed to the intervention programme 12 months after exposure	
• Adherence to recommendations on physical activity and healthy diet:	
i) % who meet the recommended level of physical activity (150 min/week of moderate physical activity or 75 min/week of intense physical activity) , among those who did not meet it at recruitment;	Baseline and 12 months after programme exposure
ii) % who meet the recommended level of fruit and vegetable intake (5 portions/day), among those who did not meet it at recruitment. •Changes in physical activity (minutes of moderate to vigorous physical activity) or in fruit and vegetable intake	
• % whose BMI decreases by 5% by 12 months after the intervention • potential effects of the preventative intervention on other cardiovascular factors including cholesterol and triglyceride levels, as well as BMI and blood glucose (data derived from the annual clinical follow-up)	Electronical Health Record

Bolded text refers to the outcome dimensions composed of multiple indicators

Underlined text refers to possible T2D risk screening strategies to be adopted by centres

Patients exposed to the intensive intervention for the promotion of healthy lifestyles (i.e. the prescription of lifestyle change) in each centre/group will be followed-up every year in the centre with measurement of clinical parameters and the assessment of physical activity and diet with the *Prescribe Vida Saludable* (Prescribe Healthy Lifestyle) questionnaires [43], incorporated in the electronic health record (Table 2).

3. Associated factors

In order to analyse the potential associated or modifying characteristics of the effect that the implementation strategy and, within it, the procedures for creating the community of practice could have on clinical practice in the primary prevention of T2D and on clinical outcomes, we studied various different factors at centre, health professional and patient levels:

- Health centre-based factors: Organisational willingness to change measured with the *Organizational Readiness for Knowledge Translation* (OR4KT) questionnaire [44], the Spanish version of the OR4KT instrument comprising 59 items assessing 6 dimensions and 23 sub-dimensions related to organisational predisposition to knowledge translation: organisational climate, organisational support, contextual factors, change content, leadership, and motivation; leadership assessed with the *Implementation Leadership Scale* (ILS) questionnaire [45]; and various characteristics of the primary care centres, namely, number of registered patients, number of healthcare professionals, mean number of registered patients per family doctor, and a socioeconomic deprivation index for the catchment area that combines variables related to employment (unemployment rate, manual and short-term employment) and education (educational attainment rate among young people and overall).
- Healthcare professional-related factors: sociodemographic variables (age, sex, professional group, etc.); and professionals’ healthy lifestyle behaviours; and Attitudes, knowledge and skills in the promotion of healthy habits in the clinical context, measured through the *Preventative Activity Questionnaire* [46].
- Patient-level factors: sociodemographic variables (age, sex, socioeconomic level- deprivation index, etc.); active health problems-morbidity (Adjusted Clinical Groups-ACG, etc.); and frequency of attendance.

Health professionals will be surveyed to obtain information related to the variables at health centre and professional level before the execution of the strategy, immediately after it and 12 months after the introduction of the programmes in the centres.

4. Fidelity of the implementation strategy

In order, on the one hand, to facilitate the replication, dissemination and future scaling of both the implementation strategy and specifically, within it, the engagement procedure used (e.g. establishment of a community of practice, execution of collaborative dynamic sessions); and on the other, to assess the degree to which what has been carried out has adhered faithfully to the plan, the process of executing the implementation strategy will be exhaustively documented and subsequent described by health centre and professional group:

- Number of centres included out of all those approached
- Percentage of health professionals who collaborate across all the centres
- Number of actions carried out (training, work sessions, etc.), and final duration compared to that originally anticipated (exposition to the implementation strategy)
- Participation of collaborating health professionals in each action
- Assessment of the content and usefulness of the actions by health professionals
- Resources allocated to the execution of the strategy in the health centres: actions and resources for the planning, organisation and execution of sessions; support materials, dedication of external facilitator, organisational support resources (freeing up of leader’s time, coverage of services, etc.)

The fidelity of the execution of the strategy and active ingredients will be assessed by triangulation of three sources of information: the protocol for the implementation strategy and the specific agendas of the working sessions; minutes, reports and other material produced in the sessions; and audio recordings of the sessions, which will be made for evaluation purposes with prior consent of the parties involved, and safeguarding the confidentiality of the information and data.

5. Qualitative evaluation

Additionally, we will carry out a qualitative evaluation of the feasibility and impact of both the strategy and the clinical intervention on the optimisation of the care practice for the primary prevention of T2D. Specifically, we will conduct semi-structured interviews with key individuals to explore the following:

- The perceived feasibility (barriers and facilitators), results observed and satisfaction of healthcare professionals, concerning the implementation strategy and the community of practice developed and the integration of the intervention programme, carrying out at least 2 interviews per participating centre (total of 18 interviews). The scripts of key-informant interviews will be theoretically-grounded, based in both the TDF and CFIR frameworks [37, 38].
- The satisfaction of users exposed to the programme with the intervention received and the impact of thereof on changes in their lifestyle habits, again carrying out at least 2 interviews per participating centre (total of 18 interviews): 1 patient who has managed to change their lifestyle habits and another who has not.

#### Management and quality of the data

All the data related to patient recruitment, the execution of the programme and the demographic characteristics of the users (age, sex, socioeconomic level) as well as the clinical variables (chronic health problems, biological and clinical parameters) will be obtained from the information extracted from the electronic health records of the Basque Health System. The Primary Care Research Unit of Bizkaia is explicitly authorised by the Healthcare Management of the Basque Health System to extract and use data from the electronic health records for research purposes. The coordination, process quality control and execution of the study, as well as management of data and ensuring its quality, are the responsibility of the Primary Care Research Unit of Bizkaia. The telephone interviews of the participants for the evaluation process of lifestyle changes will be carried out by trained professional interviewers, under the supervision of the research team. All the study data were treated anonymously and have been exclusively used for the objectives of the study. The confidentiality of personal data of participants was safeguarded at all times, in accordance with the organic law for personal data protection (15/1999 of December 13). Safety outcomes were not assessed because we did not anticipate any adverse effects due to the intervention given that it only involved physical activity and healthy diet promotion.

#### Sample size

As a worst case scenario regarding the main outcome variables for the optimisation of practices for the prevention of T2D, we hypothesise that we will obtain a rate 30% higher than a baseline level of 50% (that is, 65% in the sequential group vs 50% in the global group) in several of the outcome variables. Considering the participation of 9 centres, an alpha level of 0.05, and an intra-class correlation coefficient (ICC) at centre level of 0.03, we will need a minimum of 425 patients at high risk of developing T2D to be exposed to the prescription of a personalised plan for lifestyle change per group (850 in total) to achieve a statistical power of 80%, allowing for a possible loss of 20% of patients, in the comparison of the aforementioned process indicators related to exposure and execution of the intervention. This scenario is reasonably plausible assuming that 50% of the doctors lists in the centres agree to collaborate with this research and if we consider that, according to data from OSABIDE, an average attendance per year of 875 patients over 45 years of age with prediabetes (baseline glucose ≥110 mg/dl) per group (175 per centre). For exploring a possible modification of clinical outcomes from the prescription of lifestyle changes, namely, a 5% loss of body weight, attributable to the creation of the community of practice, we need 616 patients (308 per group) to achieve a power of 80% to reject the null hypothesis H_0_:p1 = p2 using a bilateral chi-squared test for two independent samples with a level of significance of 5%, considering that 10% of patients will attain such change in one of the groups and 35% in the other, assuming an ICC of 0.03 and a possible loss of 10% of patients at 12 months.

#### Analysis

All the analyses will be carried out on an intention-to-treat basis and then exploratory analysis will be performed by protocol and subgroup. We will estimate the absolute and relative pre-post intra-centre and between groups differences and the 95% confidence intervals using multi-level logistic regression for dichotomous variables (e.g. proportion of patients with a prescription for lifestyle change). As the sample size for evaluating the implementation objectives is over 20,000 patients, all the comparisons will be significant from a statistical point of view, and hence, emphasis will be placed on the magnitude of these differences, following the Cohen categories based on standardised differences: < 0.5; ≥ 0.5 to ≤ 0.8 and > 0.8 being considered to indicate small, moderate and large effects respectively. Comparisons will be adjusted for potential confounding factors, through stratified analysis and the inclusion of such factors in the regression models following forward and backward strategies guided by the stratified analyses.

To assess the maintenance of implementation outcomes through the 24-month follow-up, mixed-effect generalised longitudinal models will be built with fixed effects (intervention group, effect of time on monthly process indicators and interaction time-intervention) and random effects on the intercept and the slope (for each subject, doctor-centre). Said models will be adjusted for the potential confounding variables, following a forward strategy guided by the stratified analyses. Through these statistical models, we will take into account the intra-class correlation (between doctor-centre) and the hierarchical nature of data: patients nested by medical consultations and these grouped by health centre. All the analyses were carried out using SAS statistical software.

Regarding the secondary outcome variables, the percentages (e.g. % of patients who meet recommendations on physical activity) were compared using chi-square or non-parametric tests. Quantitative variables were compared using t-tests (comparison of the means). For these outcome measures, pre-post differences and differences between study groups will be estimated, and 95% confidence intervals (CIs) will be calculated on an intention-to-treat basis. Stratified analysis will be performed, mixed-effects generalised models will be built to estimate the differences between the groups adjusted for factors that confound or moderate the effect of the intervention (baseline demographic and clinical characteristics) and adjusted differences, adjusted odds ratios (ORs) and 95% CIs will be calculated at the patient level, taking into account the hierarchical and multicentre structure of data. To explore a potential modifying effect of the intervention on clinical outcomes obtained attributable to the procedure for creating the interprofessional community of practice, we will conduct comparisons by intervention protocol between groups using matching by propensity scores calculated on the basis of the characteristics of patients recorded in their health record. This will permit us to analyse the association between the actual intervention dose received (assessment of lifestyle habits, assessment of habits plus advice, and assessment of habits plus advice plus prescription) and the event of interest (change in lifestyle habits or weight loss) as a function of one or other engagement procedure, pairing patients participating in one or another comparison group in a 1:1 ratio on the basis of them having a similar probability of having received said treatment or intervention dose.

## Discussion

The present implementation research project is framed in the context of the translation of practices with proven effectiveness for the prevention of T2D. This disease is one of the main causes of illness and premature death in most countries, and hence, it has become a priority public health issue worldwide. There is an extensive evidence concerning interventions that succeed in reducing the risk of developing T2D, but there is also evidence of the gap between what it is known to be effective and what is actually being done in our health system. Yet, the implementation scientific objective is to evaluate the effect of different procedures for engaging PHC professionals in the creation of an interprofessional community of practice for the collaborative modelling and integration of a T2D primary prevention recommended clinical practice.

In a broader sense, the project seeks to integrate a T2D prevention programme, based on scientific evidence and recommended care, into the portfolio of services of PHC centres in a feasible and sustainable way, ensuring that it is as efficient as it should be, has a wide coverage and is effective among the users that could potentially benefit from it, thereby resulting in improvements in care practice, reducing the delivery of services that do not provide clinical benefits, increasing quality and improving health outcomes, through the efficient and sustainable use of health system resources. Further, the project seeks to optimise and transform clinical practice and its organisation, refocusing it towards an approach that enables feasible, sustainable and effective translation of interventions with proven efficacy, and thereby, improve the provision services by our health professionals. By incorporating and evaluating new strategies or organisational models in health centres, specifically the creation of interprofessional communities of practice, focused on collaborative planning and the redefining of professional roles, sharing out of tasks, and optimisation of the provision of care and services, will enable us to advance our knowledge of how to integrate interventions and therapies that have proven effective but are not being widely used into daily practice in sustained and continuous manner. If the findings are positive, this research will provide health planners and research community with evidence supporting the view that there is a need to introduce and assess this type of implementation strategies to enhance the adoption of new practices and innovations in operative procedures, strategies and resources, as a means to achieve integration of interventions with proven efficacy in clinical practice and in the PHC portfolio of services, and that they really reach the users who have a need for them.

However, several threats and limitations could be advanced. First, the limited number of centres in study may affect the generalizability of the study results. Second, to assure the binomial of “fidelity” in the application of the strategy and the necessary “adaptability” to the local context of each centre will be both a big challenge and an issue of scientific relevance. And third, the time necessary for the achievement of relevant changes in clinical practice is certainly unknown and also warrants careful considerations in the interpretation of study results. Additionally, low interest or lack of motivation of patients to improve their health, due to their condition of being at risk and not having a disease, may affect actual intended reach of the programmes implemented in centres. At the collaborating centre’s level, two major threats could be envisaged, being these maintaining the commitment and involvement of professionals throughout the project, and the rotation in or movement from centres of personnel. And lastly, at the organisational level, lack of support from the Directorate in the provision of resources that guarantee the correct execution of the project, and the competition with other initiatives and projects to be carried out in the organisation, may undermine implementation results.

## Abbreviations

ACG: Adjusted Clinical Groups
BMI: Body mass index
CFIR: Consolidated Framework for Implementation Research
CI: Confidence intervals
HbA1c: Haemoglobin A1c or glycated haemoglobin
ICC: Intra-class correlation coefficient
ILS: Implementation Leadership Scale
OR: Odds ratio
OR4KT: Organizational Readiness for Knowledge Translation
PHC: Primary health care
T2D: Type 2 diabetes
TDF: Theoretical Domains Framework
